# Serum interleukin-10 levels and mortality in severe fever with thrombocytopenia syndrome: a systematic review and meta-analysis

**DOI:** 10.3389/fimmu.2026.1903694

**Published:** 2026-08-07

**Authors:** Yanan Yao, Zanzan Sun, Jianguo Zhang, Zhimin Tao

**Affiliations:** 1 Department of Emergency Medicine, The Affiliated Hospital, Jiangsu University, Zhenjiang, China; 2 Jiangsu Key Laboratory of Medical Science and Laboratory Medicine, Department of Laboratory Medicine, School of Clinical Medicine and Laboratory Medicine, Jiangsu University, Zhenjiang, China

**Keywords:** biomarker, interleukin-10 (IL-10), meta-analysis, mortality, prognosis, severe fever with thrombocytopenia syndrome (SFTS)

## Abstract

**Background:**

Severe fever with thrombocytopenia syndrome (SFTS), caused by SFTS virus (SFTSV) infection, is an emerging tick-borne infectious disease associated with substantial case fatality and poses a considerable public health burden. Interleukin-10 (IL-10), an important anti-inflammatory and immunoregulatory cytokine, may reflect the magnitude of immune dysregulation in SFTS. This systematic review and meta-analysis primarily aimed to evaluate the association between serum IL-10 levels and mortality in patients with SFTSV infection. As a secondary exploratory objective, we assessed the threshold-based prognostic accuracy of IL-10 for fatal outcomes when sufficient data were available.

**Methods:**

PubMed, Embase, Web of Science, China National Knowledge Infrastructure (CNKI), Wanfang Data, and CQVIP were searched from database inception to October 11, 2025. Prospective and retrospective cohort studies, and case-control studies reporting serum IL-10 levels in survivors and non-survivors with laboratory-confirmed SFTSV infection were eligible. Two reviewers independently screened studies, extracted data, and assessed methodological quality using the Newcastle-Ottawa Scale (NOS) and risk of bias using the Quality In Prognosis Studies (QUIPS) tool for studies on prognostic factors and the Quality Assessment of Diagnostic Accuracy Studies (QUADAS)-2 tool for studies contributing threshold-based prognostic accuracy data. Standardized mean differences (SMDs) with 95% confidence intervals (CIs) were pooled using a prespecified random-effects model. Sensitivity analyses, subgroup analyses, meta-regression, funnel plots, and Egger’s test were used to evaluate the robustness of the findings and possible small-study effects.

**Results:**

Twelve studies involving 1,270 patients with SFTSV infection were included, comprising 290 non-survivors and 980 survivors. Serum IL-10 levels were significantly higher in non-survivors than in survivors (pooled SMD = 2.08, 95% CI: 1.40-2.76; P<0.01). Substantial heterogeneity was observed (*I*^2^ = 94%, P<0.01), and the 95% prediction interval crossed zero. Sensitivity analyses generally preserved the direction of the association, including an analysis restricted to studies not requiring conversion from medians and quantiles; however, substantial residual heterogeneity and the limited number of directly reported datasets indicated considerable uncertainty regarding the magnitude of the pooled effect. Subgroup analyses according to study design showed a directionally consistent association, whereas exploratory meta-regression found no statistically significant relationship between study-level IL-10 concentration and effect size. Funnel-plot asymmetry and Egger’s regression test indicated possible small-study effects; however, the extreme between-study heterogeneity limited the ability to distinguish selective publication from methodological or clinical variability. Threshold-based prognostic-accuracy analyses suggested potential discriminatory value for mortality prediction, but they were based on only three studies using *post hoc*, non-validated cut-off values and therefore remained exploratory. The certainty of evidence for the association between IL-10 levels and mortality was rated as very low because of residual confounding, inconsistency, indirectness, and possible small-study effects.

**Conclusion:**

Elevated serum IL-10 levels were associated with mortality in SFTS; however, the certainty of evidence was very low. IL-10 should therefore be considered a candidate prognostic biomarker requiring further prospective validation rather than a clinically established stand-alone prognostic tool.

**Systematic review registration:**

https://www.crd.york.ac.uk/prospero/, identifier CRD420251149587.

## Introduction

Severe fever with thrombocytopenia syndrome (SFTS) is an emerging tick-borne zoonotic disease caused by SFTS virus (SFTSV) (1). It is clinically characterized by high fever, thrombocytopenia, leukopenia, gastrointestinal symptoms, and, in severe cases, multi-organ dysfunction (2). Since its first recognition in rural China in 2009, SFTS has been reported in several other countries, including South Korea, Vietnam and Japan (3). Despite advances in clinical virology and disease management, the pathogenesis of SFTS remains incompletely understood, and no specific antiviral therapy has been universally established (4). Therefore, early identification of patients at high risk of fatality is essential for timely clinical intervention and risk stratification.

The pathophysiology of SFTSV infection involves direct viral injury together with profound host immune dysregulation (5). Following viral invasion, excessive activation of innate and adaptive immune responses can lead to the release of pro-inflammatory cytokines, endothelial injury, coagulation dysfunction, and ultimately multiple organ dysfunction syndrome (MODS). A cytokine storm has been proposed as an important driver of severe disease progression (6, 7). However, the immune response in SFTS is not limited to pro-inflammatory activation. Dysregulated anti-inflammatory responses may also contribute to viral persistence, immune paralysis, and poor outcomes (8).

Interleukin-10 (IL-10) is a potent anti-inflammatory cytokine produced by multiple immune cell types, including regulatory T cells, monocytes, macrophages, and dendritic cells (9). Under physiological conditions, IL-10 limits inflammatory injury by suppressing the production of pro-inflammatory cytokines. During acute viral infections, however, excessive IL-10 production may inhibit antiviral cellular immune responses and impair viral clearance, whereas markedly elevated levels may also reflect a compensatory response to uncontrolled systemic inflammation and thus indicate augmented disease severity (10, 11). Several other biomarkers, including IL-6, TNF-α, ferritin, viral load, procalcitonin, and serum calcium, have also been investigated as indicators of severity or mortality in SFTS (8, 12–15). In contrast to predominantly pro-inflammatory mediators such as IL-6 and TNF-α, IL-10 reflects a distinct immunoregulatory component of the host response. Moreover, the SFTSV nonstructural protein NSs has been reported to promote IL-10 production through activation of the TPL2 signaling pathway, providing a potential mechanistic basis for its association with adverse clinical outcomes (16). IL-10 may therefore offer complementary, rather than superior, prognostic information alongside other inflammatory and virological indicators.

In recent years, several clinical studies have observed the close relationship between serum IL-10 levels and outcomes in patients with SFTSV infection (14, 17–19). Studies have reported significantly higher IL-10 levels in non-survivors than in survivors, suggesting a potential association with poor prognosis. Nevertheless, the reliability and generalizability of these findings remain uncertain due to the small number of participants, differences in study design, variable patient characteristics, and inconsistent IL-10 measurement methods. To our knowledge, evidence regarding serum IL-10 and mortality in SFTS has not been comprehensively assessed. Therefore, the primary objective of this systematic review and meta-analysis was to determine whether serum IL-10 levels differed between survivors and non-survivors with laboratory-confirmed SFTSV infection. A secondary exploratory objective was to summarize the available evidence regarding the threshold-based prognostic accuracy of IL-10 for mortality. Because an inter-group difference in biomarker concentration does not necessarily indicate adequate individual-level discrimination, the association and threshold-based prognostic accuracy analyses were interpreted separately.

## Methods

### Literature search and screening

A systematic search of PubMed, Embase, Web of Science, China National Knowledge Infrastructure (CNKI), Wanfang Data, and CQVIP was conducted from database inception to October 11, 2025. Database-specific strategies combined controlled vocabulary, including MeSH or Emtree terms where applicable, with free-text terms for “severe fever with thrombocytopenia syndrome”, “SFTS”, “SFTSV”, “bunyavirus”, “novel bunyavirus”, “interleukin-10”, and “IL-10”. Searches were restricted to studies published in English or Chinese. Reference lists of all eligible studies and relevant reviews were manually screened for additional records. Conference abstracts without sufficient extractable data were excluded. Preprint servers and other grey-literature databases were not systematically searched. Boolean operators, field tags, truncation rules, and the complete database-specific search strategies are provided in the **Supplementary Materials**.

All retrieved records were imported into EndNote X9.3 for management. Duplicates were first removed using the “Find Duplicates” function and then verified by two independent reviewers, including overlapping conference abstracts and full-text publications. Inclusion criteria were as follows: (1) studies involving patients with laboratory-confirmed SFTSV infection; (2) studies reporting serum IL-10 levels in both survivor and non-survivor groups; and (3) studies providing sufficient data for extraction or conversion, including mean ± standard deviation (SD), median with interquartile range (IQR), or median with range. Exclusion criteria included: (1) abstracts without full-text data, case reports with fewer than five patients, letters, editorials, reviews, *in vitro* studies, animal studies, genotypic studies, and treatment-only studies; (2) studies with incomplete or non-extractable IL-10 data; and (3) duplicate or overlapping reports, in which case only the most complete dataset was retained.

The protocol registered in PROSPERO (CRD420251149587) pre-specified the following analyses: pooling of SMDs, heterogeneity assessment using *I*^2^ and Q tests, subgroup analysis by study design, sensitivity analysis, and Egger’s test for possible small-study effects. The following analyses were performed *post hoc* and are explicitly designated as exploratory: (1) meta-regression examining the relationship between study-level IL-10 concentration and mortality effect size; and (2) threshold-based prognostic-accuracy analysis, including sensitivity, specificity, likelihood ratios, summary receiver operating characteristic (SROC) curves, area under the curve (AUC), and a Fagan nomogram, after three studies with sufficient dichotomous data were identified.

### Data extraction and quality assessment

Two reviewers independently extracted data from the included studies using a predefined data extraction form. Disagreements were resolved by discussion or, when necessary, by consultation with a third reviewer. The extracted information included: first author, year of publication, country or region, study design, sample size, numbers of survivors and non-survivors, serum IL-10 levels in each group, measurement units and assay methods when available, and information required for risk-of-bias assessment. For studies reporting IL-10 concentrations as medians with IQRs or ranges, means and SDs were estimated using the reported method (20, 21). Eight studies required this conversion: Yoo et al. (2021) (18), Zhang et al. (2012) (22), Liu et al. (2025) (23), Lu et al. (2024) (24), Ma et al. (2020) (25), Zang et al. (2024) (26), Zhang et al. (2024) (27), and Yu et al. (2025) (28). In addition, Li et al. (2014) (29) reported means and standard errors, from which SDs were calculated using the formula SD=SE×n^1/2^ (30). Hou et al. (2024) (31), Zhao et al. (2020) (32), and Liu et al. (2020) (33) directly reported means and SDs. The original data format and conversion procedure for each study are presented in **Supplementary Table 1**. Only one study, Li et al. (2014), reported serial IL-10 measurements and adopted the highest concentration recorded between days 5 and 15 after symptom onset. The remaining eleven studies took a single measurement obtained at hospital admission or during early hospitalization. Because peak cytokine concentrations are not comparable with baseline measurements, combining these sampling strategies may introduce additional clinical heterogeneity and potentially influence the pooled effect estimate.

The methodological quality of cohort and case-control studies was assessed using the NOS (34, 35). The NOS evaluates selection of study groups, comparability of groups, and ascertainment of exposure or outcome. Risk of bias in studies evaluating IL-10 as a prognostic factor was assessed using the QUIPS tool. QUIPS evaluates six domains: study participation, study attrition, prognostic factor measurement, outcome measurement, study confounding, and statistical analysis and reporting. For studies contributing threshold-based prognostic accuracy data, the QUADAS-2 tool was additionally applied across the domains of patient selection, index test, reference standard, and flow and timing. QUIPS domains were rated as low, moderate, or high risk of bias, whereas QUADAS-2 domains were rated as low, high, or unclear risk of bias.

The certainty of evidence for the principal outcomes was evaluated using a Grading of Recommendations Assessment, Development and Evaluation (GRADE) framework adapted for prognostic factor assessment. The assessment considered risk of bias, inconsistency, indirectness, imprecision, and possible small-study effects. Certainty was categorized as high, moderate, low, or very low. All assessments were performed independently by two reviewers, with disagreements resolved through discussion or consultation with a third reviewer.

### Statistical analysis

Statistical analyses were performed using RevMan 5.4, StataMP 18, MetaDiSc 1.4, and RStudio. For effect size pooling, since IL-10 levels were measured using different methods and may have been reported in different units across studies, the standardized mean difference (SMD) was selected as the primary effect measure. Pooled effect estimates are presented with 95% confidence intervals (CIs). Given the anticipated clinical and methodological diversity across the included studies, including differences in patient populations, disease severity, timing of blood sampling, assay platforms, and study design, a random-effects model using the DerSimonian-Laird method was specified *a priori* as the primary analytical approach, irrespective of the observed heterogeneity statistics.

Between-study heterogeneity was assessed using Cochran’s Q test and quantified using the *I*² statistic and τ². Fixed-effect estimates were calculated only as sensitivity analyses. Potential sources of heterogeneity were explored using subgroup and sensitivity analyses. The principal subgroup analysis was conducted according to study design, categorized as prospective cohort, retrospective cohort, or case-control study. The classification according to study-level IL-10 concentration was not based on validated biological or clinical thresholds and was presented only in the **Supplementary Materials** as a *post hoc* exploratory analysis.

Sensitivity analysis was performed by sequentially excluding one study at a time to evaluate whether any individual study substantially influenced the pooled estimate. Potential small-study effects were evaluated by visual inspection of funnel plots and Egger’s regression test. Because funnel-plot asymmetry may arise from publication bias, between-study heterogeneity, methodological differences, or other sources, it was not interpreted as definitive evidence of selective publication. A trim-and-fill analysis was additionally performed as an exploratory sensitivity analysis, with recognition that its reliability may be limited in the presence of substantial heterogeneity. A univariable random-effects meta-regression was performed as an exploratory analysis to examine whether study-level IL-10 concentration was associated with the SMD between non-survivors and survivors. Because absolute IL-10 concentrations varied markedly across studies, IL-10 concentration was log-transformed before analysis.

## Results

### Literature screening process and overview of included studies

**Figure 1:**
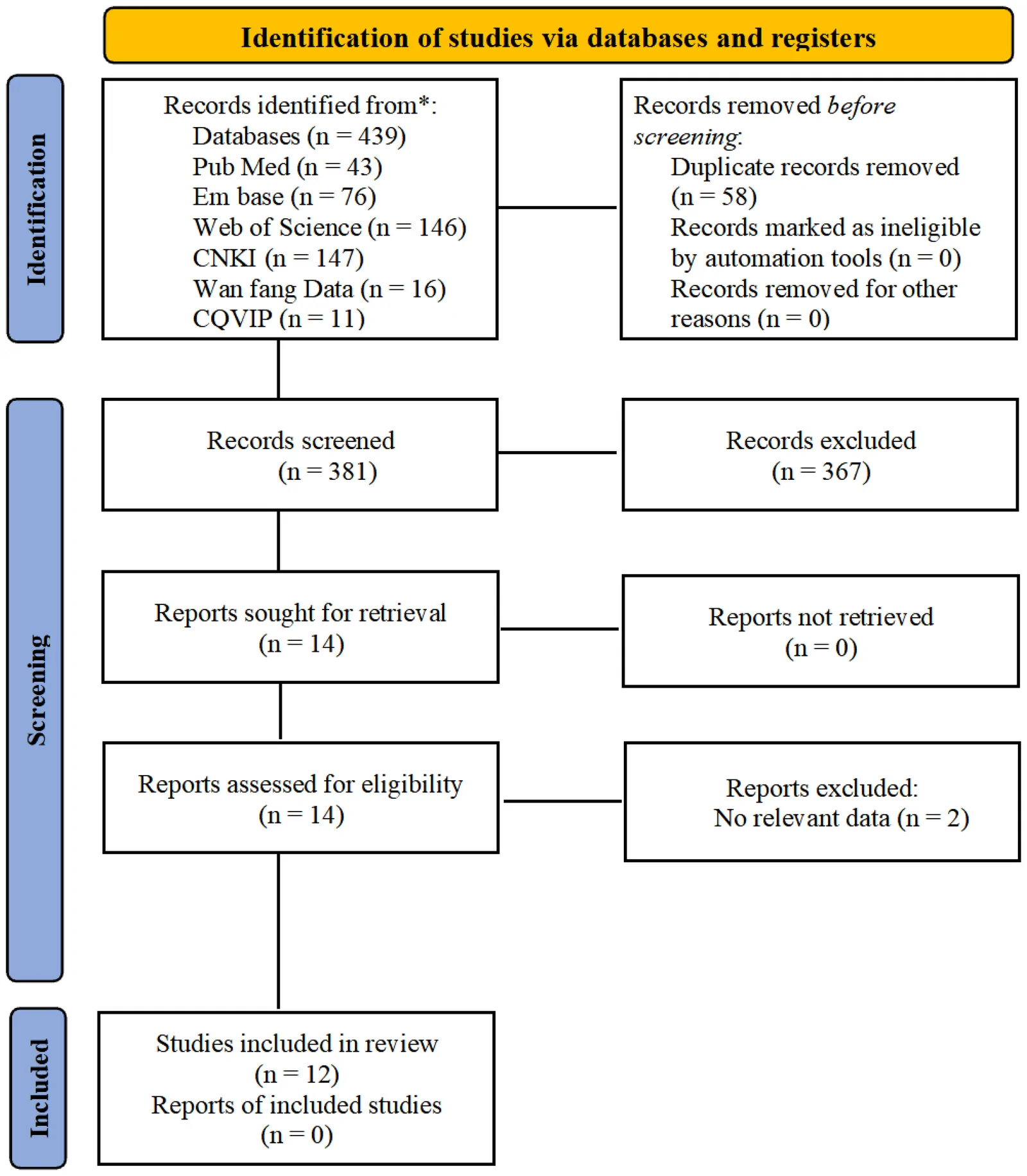
PRISMA flow diagram of study selection. A total of 439 records were identified from six databases: PubMed, Embase, Web of Science, China National Knowledge Infrastructure (CNKI), Wanfang Data, and CQVIP. After removal of 58 duplicate records, 381 records remained for title and abstract screening, of which 367 were excluded. Fourteen full-text articles were assessed for eligibility, and two were excluded because they did not provide extractable IL-10 data. Finally, 12 studies were included.

The initial database search identified 439 records. After removal of duplicates, 381 records remained. Screening of titles and abstracts excluded 367 clearly irrelevant records. Fourteen full-text articles were assessed for eligibility, of which two were excluded because they did not provide relevant extractable data. As a result, twelve studies met the inclusion criteria and were included in the meta-analysis. The included studies were published between 2011 and 2025 and involved 1,270 patients with SFTSV infection, consisting of 290 non-survivors and 980 survivors. The studies were mainly conducted in China and South Korea and contained four prospective cohort studies (18, 22, 23, 29), seven retrospective cohort studies (24–28, 31, 33), and one case-control study (32). Details regarding the biological sample matrix, detection method, measurement unit, and main findings for each study are summarized in **Supplementary Table 2** of **Supplementary Materials**. The literature screening process followed PRISMA guidance and was illustrated in **Figure 1**.

### Risk-of-bias assessment

According to NOS assessment, the included studies scored between 6 and 8 points, indicating generally moderate-to-high methodological quality. The QUIPS assessment indicated an overall moderate risk of bias for all 12 studies included in the prognostic factor analysis. The studies were generally judged to have a low risk of bias in study participation, outcome measurement, and statistical analysis and reporting. However, prognostic factor measurement was rated as moderate risk in five studies: four because the IL-10 assay method was not reported and one because peak IL-10 concentrations obtained during days 5–15 after symptom onset were not directly comparable with admission or early hospitalization measurements. Study confounding remained the principal concern and was rated as moderate risk in all studies. Detailed domain-level judgments are presented in **Supplementary Figure 1**. The three studies contributing to the threshold-based prognostic accuracy analysis were assessed using QUADAS-2. These studies were judged to have a low risk of bias in patient selection, reference-standard classification, and flow and timing, but a high risk of bias in the index-test domain because the IL-10 cut-off values were derived *post hoc* using receiver operating characteristic analyses rather than being prespecified or externally validated. Consequently, the overall risk of bias was rated as high for all three studies. Detailed results are presented in **Supplementary Figure 2**.

Furthermore, the certainty of evidence for the association between higher serum IL-10 levels and mortality was rated as very low. The evidence was downgraded because of risk of bias arising primarily from inadequate adjustment for prognostic confounders, extreme inconsistency (*I*^2^ = 94% and a prediction interval crossing zero), indirectness related to assay and sampling variability and geographic concentration of the evidence, and concern regarding possible small-study effects.

### Overall effect analysis of IL-10 levels and mortality rates

**Figure 2:**
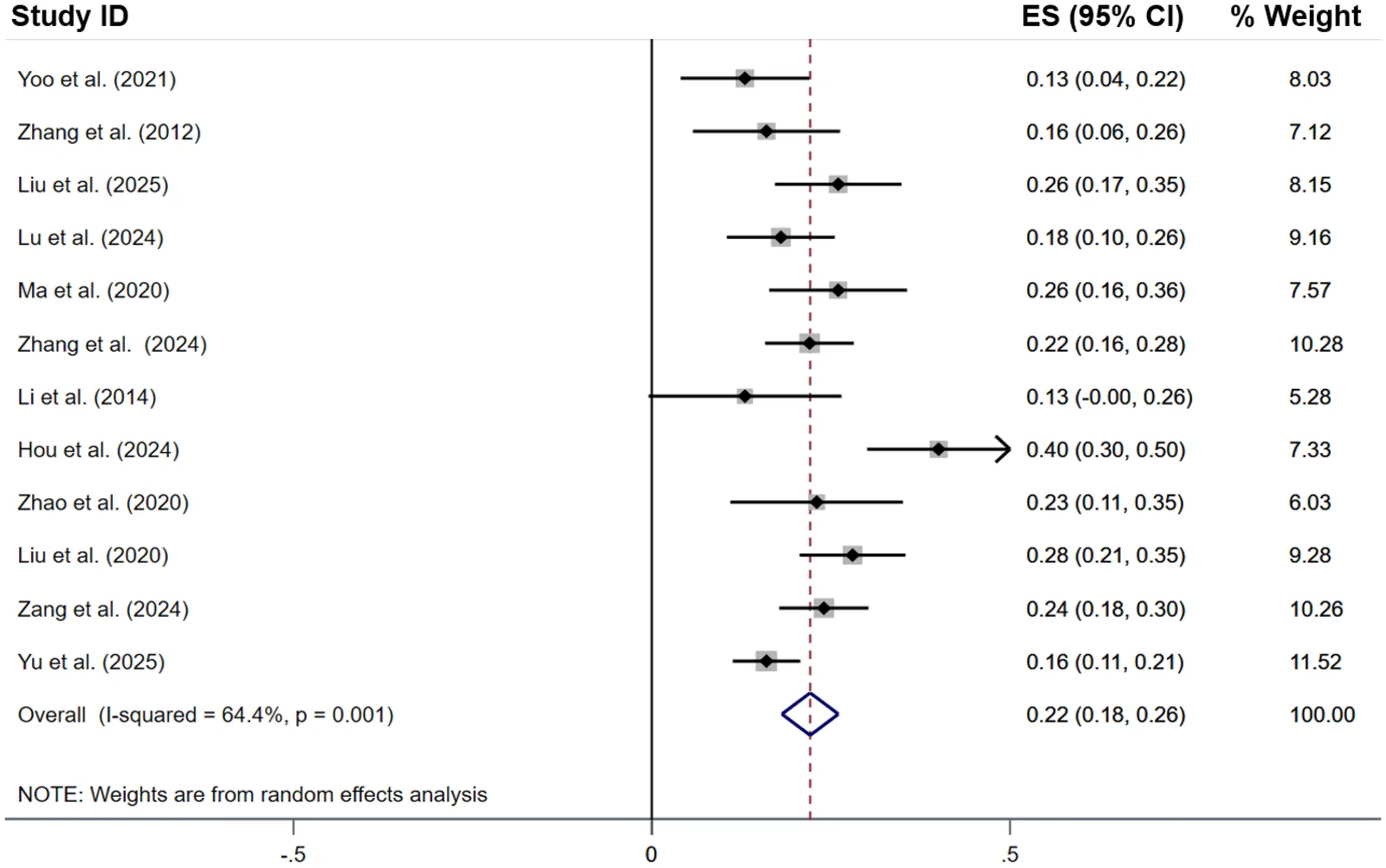
Forest plot of the mortality proportion among patients with SFTS in the included studies. Squares represent the study-specific mortality proportions, horizontal lines indicate the corresponding 95% confidence intervals (CIs), and square size reflects the weight assigned in the random-effects model. The diamond represents the pooled mortality proportion and its 95% CI. The pooled mortality proportion was 22% (95% CI: 18%-26%), with substantial between-study heterogeneity (*I*^2^ = 64.4%, P = 0.001). The arrow indicates that the upper confidence limit extends beyond the displayed axis range. CI, confidence interval; ES, effect size.

**Figure 3:**
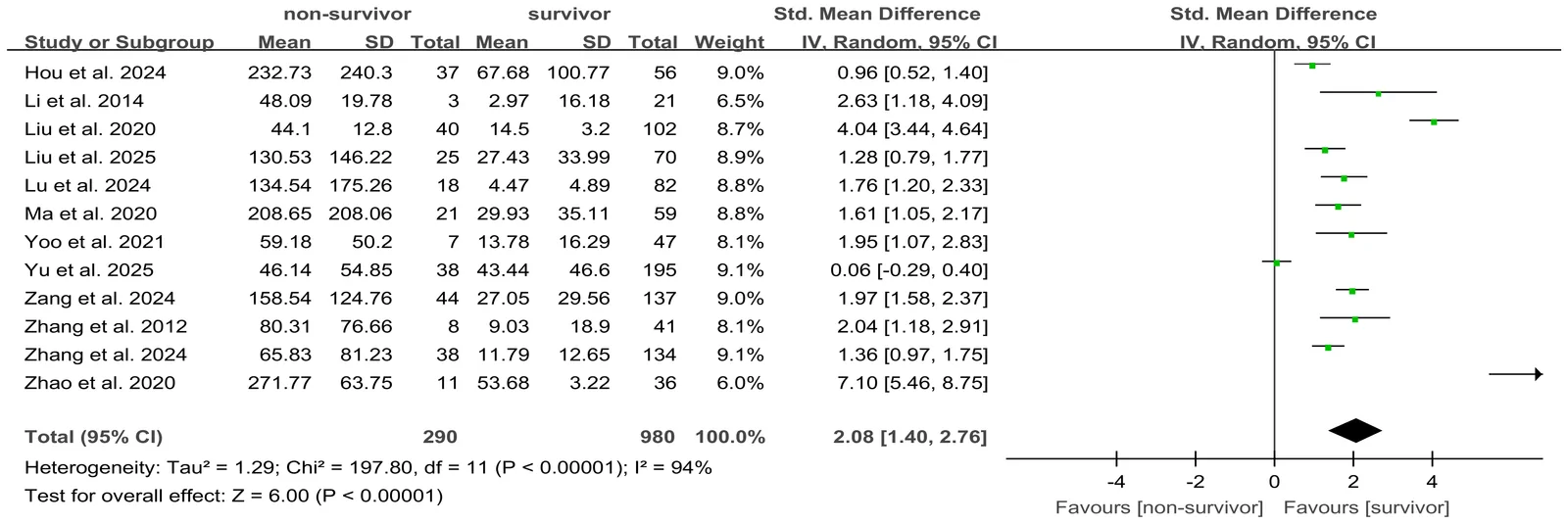
Forest plot comparing serum IL-10 levels between non-survivors and survivors with SFTS. Squares represent study-specific standardized mean differences (SMDs; Hedges’ *g*), horizontal lines indicate the corresponding 95% confidence intervals (CIs), and square size reflects the weight assigned under the DerSimonian-Laird random-effects model. The diamond represents the pooled effect estimate. Serum IL-10 levels were significantly higher in non-survivors than in survivors (pooled SMD = 2.08, 95% CI:1.40-2.76), with substantial between-study heterogeneity (*I*^2^ = 94%, P<0.001). Zhao et al. (2020) (32) reported an exceptionally large effect estimate (SMD = 7.10), whereas Yu et al. (2025) (28) reported an effect estimate close to zero (SMD = 0.06). CI, confidence interval; SMD, standardized mean difference.

Among the 1,270 patients included in the 12 selected studies, 290 died, corresponding to a crude mortality proportion of 22% (95% CI: 0.18-0.27; **Figure 2**). Because study eligibility required reporting IL-10 levels according to survival status, this proportion is descriptive of the included dataset and should not be interpreted as a representative population-level case-fatality estimate for SFTS. Using the prespecified random-effects model, the pooled SMD was 2.08 (95% CI: 1.40-2.76; P<0.01; **Figure 3**) with substantial between-study heterogeneity (*I*²= 94%; τ² = 1.29; P<0.01). The 95% prediction interval ranged from -0.66 to 4.62 and crossed zero, indicating that the effect observed in a future study may range from a negative or null association to a strong positive association under different clinical and methodological settings. Although the pooled association was statistically significant, the very low certainty of evidence indicates that the true effect may differ substantially from the pooled estimate. Thus, although the average pooled effect suggests higher IL-10 levels among non-survivors, the magnitude and consistency of this association remain uncertain. The pooled estimate was based predominantly on unadjusted differences in IL-10 concentrations and should therefore be interpreted as an association rather than evidence that IL-10 is an independent prognostic factor.

**Figure 4:**
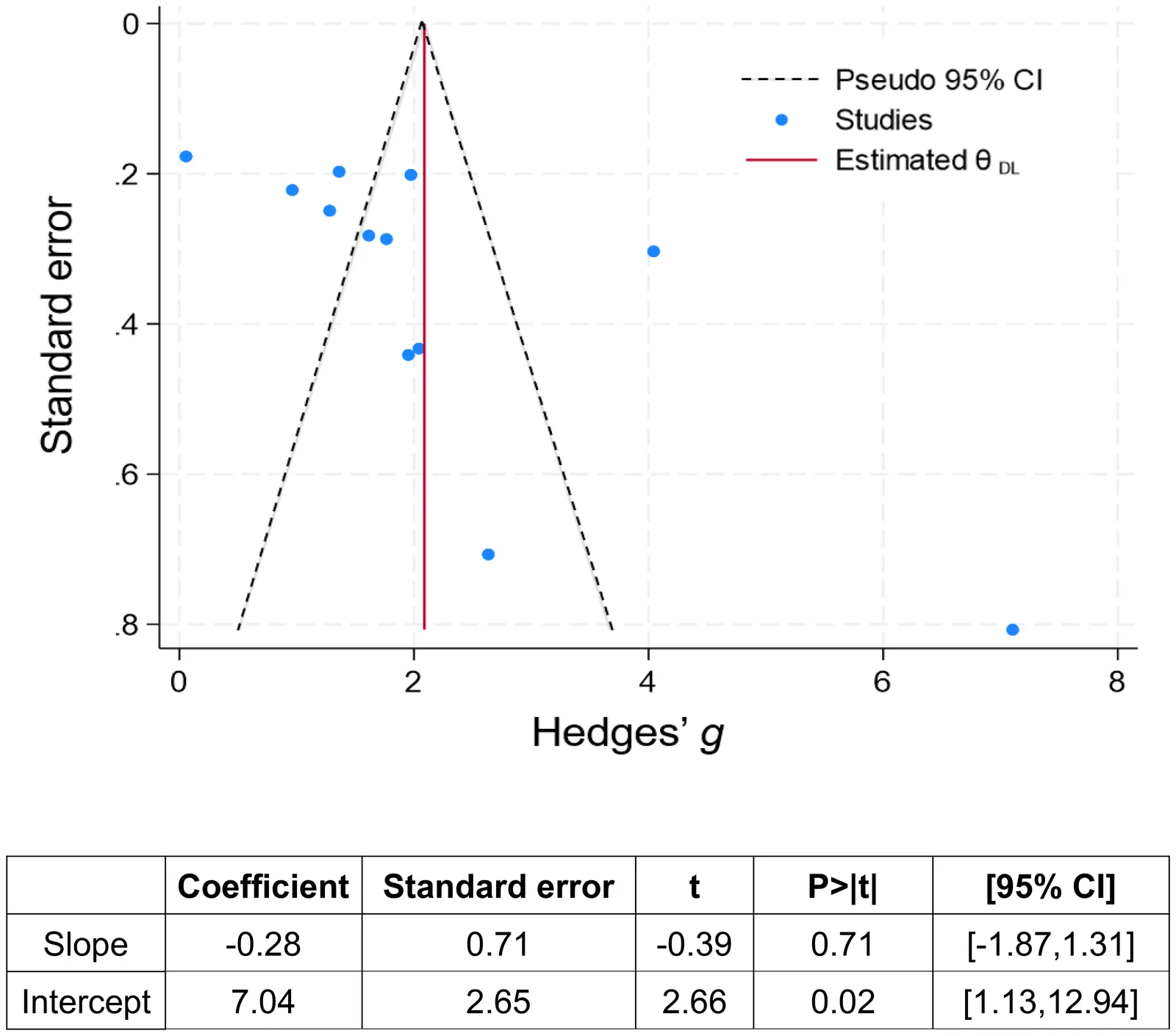
Assessment of funnel-plot asymmetry and possible small-study effects. Each dot represents an individual study included in the meta-analysis comparing serum IL-10 levels between survivors and non-survivors. The horizontal axis shows the standardized mean difference (SMD), and the vertical axis shows its standard error (SE), with more precise studies appearing toward the top. The vertical solid line indicates the pooled SMD, and the diagonal dashed lines represent the pseudo 95% confidence limits. Visual inspection suggested funnel-plot asymmetry, and Egger’s regression test yielded a statistically significant intercept (intercept = 7.04, 95% CI: 1.13-12.94; *P* = 0.02). However, given the substantial between-study heterogeneity, this finding should be interpreted as evidence of possible small-study effects rather than definitive publication bias. CI, confidence interval; SE, standard error; SMD, standardized mean difference.

Visual inspection indicated funnel-plot asymmetry, and Egger’s regression test suggested possible small-study effects (intercept = 7.04, P = 0.02; **Figure 4**). However, because between-study heterogeneity was extreme (*I*^2^ = 94%), the asymmetry may also reflect genuine clinical or methodological variability and should not be interpreted as definitive evidence of publication bias. In the exploratory trim-and-fill analysis conducted on the SMD scale, no potentially missing studies were imputed, and the pooled estimate remained unchanged at an SMD of 2.11 (95% CI: 1.42-2.79), with persistent heterogeneity (Q = 202.38, df=11, P<0.01; **Supplementary Figure 3**). However, the absence of imputed studies does not exclude publication bias or small-study effects, particularly given the limited number of included studies and the extreme between-study heterogeneity. These findings should therefore be interpreted cautiously.

### Sensitivity analysis

Two studies showed notably divergent effect estimates. Zhao et al. (2020) (32) reported an exceptionally large SMD of 7.10, whereas Yu et al. (2025) (28) reported an SMD of 0.06. We rechecked the original publications and verified the extracted values, sample sizes, units, and data conversion procedures. In Zhao et al., the large SMD was primarily attributable to the very small standard deviation reported in the survivor group (53.68 ± 3.22 pg/mL). In Yu et al. (2025), IL-10 was reported in the unit of ng/mL, and the concentrations were nearly identical between non-survivors and survivors. Conversion to pg/mL changed the absolute numerical scale but did not affect the SMD because the same conversion factor was applied to both groups.

**Figure 5:**
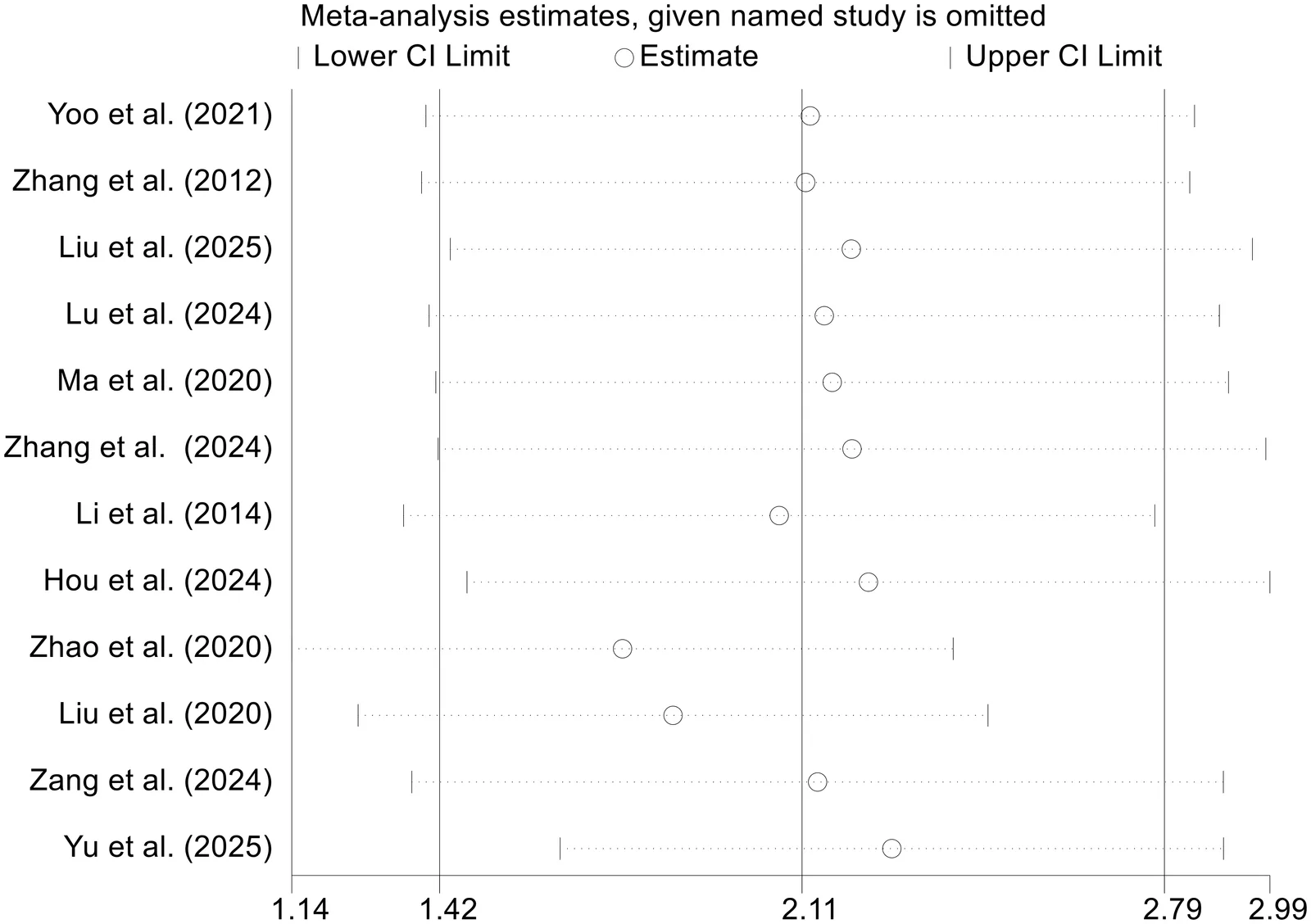
Leave-one-out sensitivity analysis of the association between serum IL-10 levels and mortality in SFTS. Each row presents the recalculated pooled standardized mean difference (SMD), expressed as Hedges’ *g*, and its corresponding 95% confidence interval (CI) after omission of the indicated study. Open circles represent the pooled estimates, and the short vertical marks indicate the lower and upper 95% CI limits. The central vertical line represents the overall pooled effect estimate from the complete dataset, while the two flanking vertical lines indicate its 95% CI. Exclusion of any single study did not alter the direction or statistical significance of the association, as all recalculated estimates remained positive and none of the 95% CIs crossed zero. However, the magnitude of the pooled effect varied across the analyses, indicating directional robustness but continued uncertainty regarding the precise effect size. CI, confidence interval; SMD, standardized mean difference.

**Figure 6:**
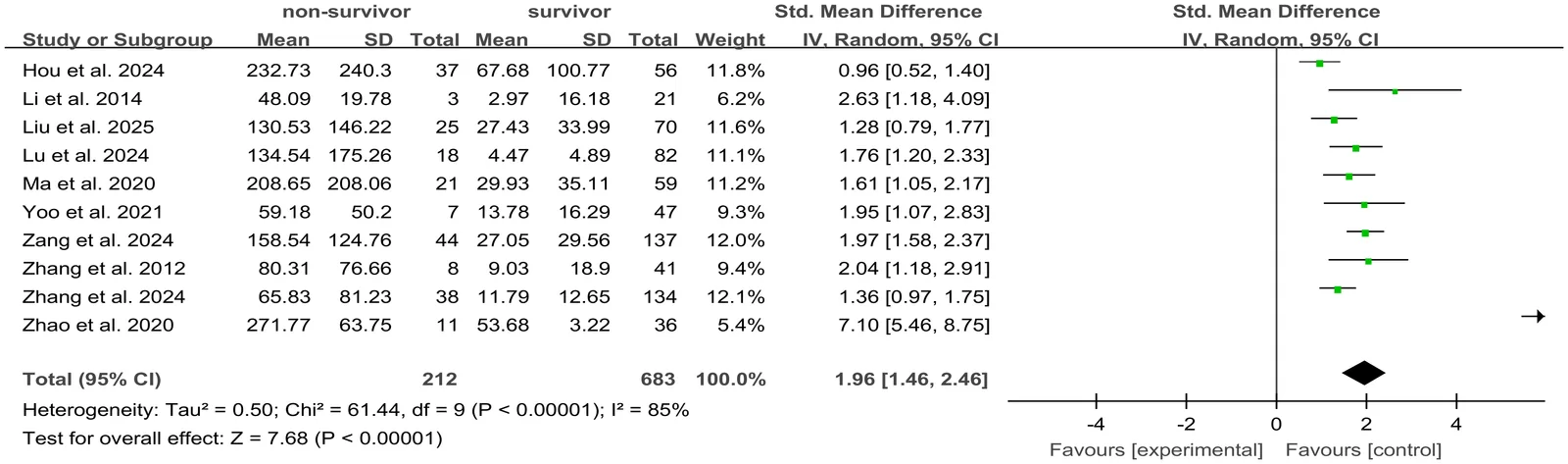
Forest plot of the meta-analysis after exclusion of two influential studies. Squares represent study-specific standardized mean differences (SMDs; Hedges’ *g*), horizontal lines indicate the corresponding 95% confidence intervals (CIs), and square size reflects the weight assigned under the DerSimonian-Laird random-effects model. The diamond represents the pooled effect estimate. After exclusion of the two influential studies, the pooled association remained statistically significant and directionally consistent (SMD = 1.96, 95% CI:1.46-2.46). However, substantial heterogeneity persisted (*I*^2^ = 85%, P<0.001), indicating continued uncertainty regarding the magnitude of the association. CI, confidence interval; SMD, standardized mean difference.

Sensitivity analysis was performed by excluding each study sequentially. The overall direction of the association remained unchanged across most analyses (**Figure 5**). Two studies, Yu et al. (2025) (28) and Liu et al. (2020) (33), showed CIs with limited overlap with the overall pooled estimate. After excluding these two studies, the pooled association remained statistically significant and directionally consistent (SMD = 1.96, 95% CI: 1.46-2.46; **Figure 6**); however, substantial heterogeneity persisted (*I*^2^ = 85%), indicating that the magnitude of the association remained uncertain.

An additional sensitivity analysis was performed after excluding the eight studies for which means and SDs had been estimated from medians with IQRs or ranges. The analysis was therefore restricted to Hou et al. (2024) (31), Zhao et al. (2020) (32), Liu et al. (2020) (33), and Li et al. (2014) (29), the latter of which reported means and SEs and allowed direct calculation of SDs. The pooled SMD was 3.61 (95% CI: 1.29-5.93), and substantial heterogeneity persisted (*I*^2^ = 97%, τ^2^ = 5.29). Although the direction of the association remained consistent with the primary analysis, the magnitude of the estimate was highly uncertain because only four studies were included. The corresponding forest plot is presented in (**Supplementary Figure 4**).

### Subgroup analysis

**Figure 7:**
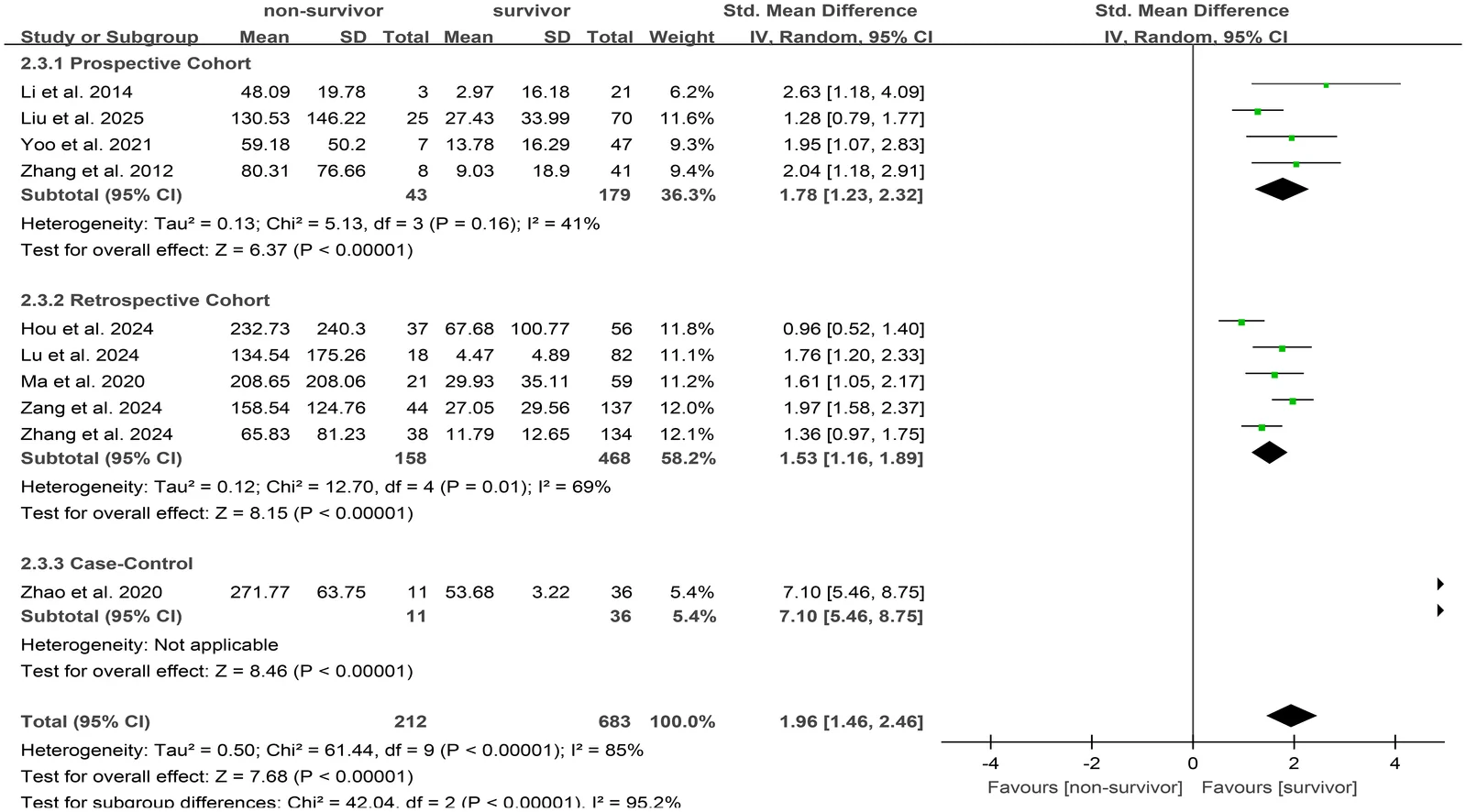
Subgroup analysis according to study design. Forest plot showing the association between serum IL-10 levels and mortality in patients with SFTS, stratified by study design after exclusion of two influential studies. Squares represent study-specific standardized mean differences (SMDs; Hedges’ *g*), horizontal lines indicate the corresponding 95% confidence intervals (CIs), and square size reflects the weight assigned under the DerSimonian-Laird random-effects model. Diamonds represent the pooled estimates for each subgroup and for the overall analysis. The pooled SMD was 1.78 (95% CI: 1.23-2.32; *I*^2^ = 41%) for four prospective cohort studies, 1.53 (95% CI:1.16-1.89; *I*^2^ = 69%) for five retrospective cohort studies, and 7.10 (95% CI: 5.46-8.75) for the single case-control study. The overall pooled SMD was 1.96 (95% CI: 1.46-2.46), with substantial heterogeneity (*I*^2^ = 85%). CI, confidence interval; SMD, standardized mean difference.

To investigate potential sources of heterogeneity, subgroup analyses were conducted according to study design. In the sensitivity dataset after exclusion of the two influential studies [i.e., Yu et al., 2025 (28) and Liu et al., 2020 (33)] (**Figure 7**), the pooled SMD was 1.78 (95% CI:1.23-2.32) among four prospective cohort studies, with moderate heterogeneity (*I*^2^ = 41%). Five retrospective cohort studies remained the pooled SMD of 1.53 (95% CI: 1.16-1.89), with moderate heterogeneity (*I*^2^ = 69%). The case-control subgroup contained only Zhao et al. (2020) (32), which reported an SMD of 7.10 (95% CI: 5.46-8.75). Therefore, this value represents an individual study estimate rather than a pooled subgroup effect. These findings suggest that the positive association was directionally consistent across cohort designs, although the magnitude of the association remained variable.

The *post hoc* subgroup analysis according to study-level IL-10 concentration is presented in **Supplementary Figure 5**. Because the categories were derived from the distribution of study-level concentrations and were not based on validated biological or clinical thresholds, the analysis was considered exploratory. The test for subgroup differences was not statistically significant (P = 0.46), providing no evidence that these concentration categories may explain the observed heterogeneity. Therefore, any variation among subgroup-specific estimates should be regarded as hypothesis-generating only. Differences in the timing of blood sampling may have also contributed to the observed heterogeneity, as one study contributed peak IL-10 concentrations from serial measurements, whereas the remaining studies reported values measured at hospital admission or early hospitalization time.

### Exploratory meta-regression analysis

A meta-regression analysis was used to explore the relationship between IL-10 concentration and mortality-related effect size (**Supplementary Figure 6**). Exploratory univariable meta-regression showed no statistically significant linear association between log-transformed study-level IL-10 concentration and the mortality-related effect size (*β* = 0.73, SE = 0.96; *t* = 0.76; 95% CI: −1.48-2.93; P = 0.47). The intercept was −1.28 (SE = 4.60; *t*=−0.28; 95% CI: −11.90-9.33; P = 0.79). Because only 10 studies (after exclusion of two influential studies as mentioned above) were included and the result was sensitive to extreme observations, this analysis was considered exploratory.

### Prognostic accuracy of IL-10 for mortality

**Figure 8:**
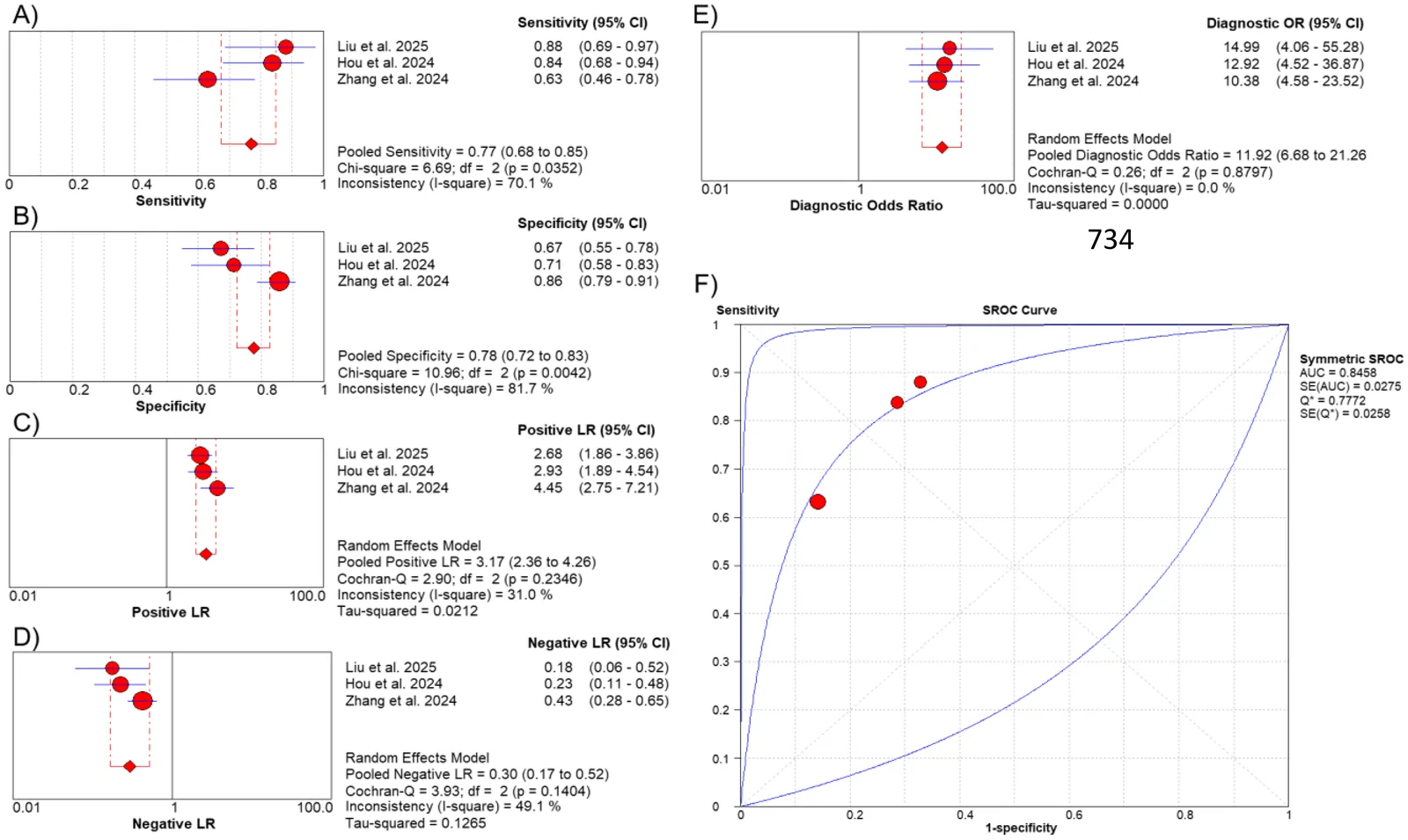
Exploratory analysis of the threshold-based prognostic accuracy of IL-10 for fatal outcomes in SFTS. The figure summarizes the sensitivity **(A)**, specificity **(B)**, positive likelihood ratio (LR+) **(C)**, negative likelihood ratio (LR−) **(D)**, and diagnostic odds ratio (DOR) **(E)** derived from three studies: Liu et al. (2025) (23), Hou et al. (2024) (31), and Zhang et al. (2024) (27). Summary estimates were obtained using separate univariate random-effects models. The pooled sensitivity was 77% (95% CI: 68%-85%), specificity was 78% (95% CI: 72%-83%), LR+ was 3.17 (95% CI: 2.36-4.26), LR− was 0.30 (95% CI: 0.17-0.52), and DOR was 11.92 (95% CI: 6.68-21.26). **(F)** The summary receiver operating characteristic (SROC) curve yielded an AUC of 0.85 (SE = 0.0275), suggesting moderate discriminatory performance. Because only three studies with heterogeneous, data-derived thresholds were included, all summary estimates should be considered exploratory. AUC, area under the curve; CI, confidence interval; DOR, diagnostic odds ratio; LR, likelihood ratio; SE, standard error; SROC, summary receiver operating characteristic.

Three studies provided data suitable for evaluating the threshold-based prognostic accuracy of IL-10 for fatal outcomes (**Figure 8**). The IL-10 cut-offs were 45.2 pg/mL in Liu et al. (2025) (23), 42.62 ng/L in Zhang et al. (2024) (27), and 112.5 pg/mL in Hou et al. (2024) (31). All thresholds were data-derived using ROC analyses rather than prespecified or externally validated. The pooled sensitivity was 77% (95% CI: 0.68-0.85), the pooled specificity was 78% (95% CI: 0.72-0.83), the pooled positive likelihood ratio (LR) was 3.17 (95% CI: 2.36-4.26), and the pooled negative LR was 0.30 (95% CI: 0.17-0.52). The pooled diagnostic odds ratio was 11.92 (95% CI: 6.68-21.26), and the area under the receiver operating characteristic (AUROC) curve was 0.85. Because these estimates were derived from only three studies using *post hoc*, non-validated thresholds, the pooled AUROC and likelihood ratios are statistically unstable and should not be interpreted as evidence supporting clinical implementation or routine risk stratification.

**Figure 9:**
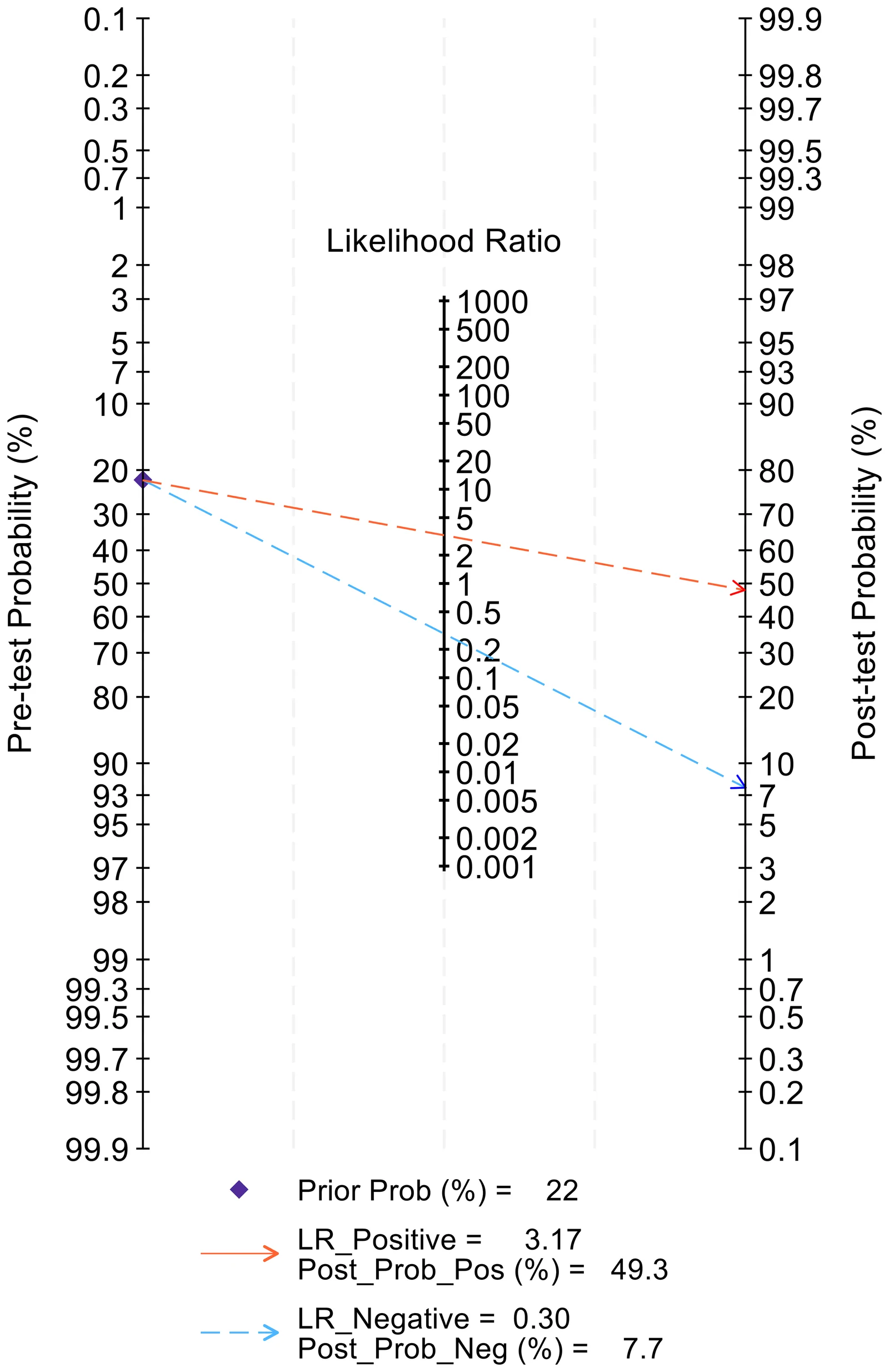
Illustrative Fagan nomogram showing pre-test and post-test probabilities for fatal outcomes based on IL-10. Using a pre-test probability of 22%, derived from the mortality proportion among the included studies, a positive IL-10 result increased the post-test probability of fatal outcome to 49.3%, whereas a negative result decreased it to 7.7%. The nomogram was based on a positive likelihood ratio of 3.17 and a negative likelihood ratio of 0.30. Because the pre-test probability was derived from all included studies rather than specifically from the three studies contributing to the prognostic accuracy analysis, and because post-test probabilities are prevalence-dependent, these estimates are presented for illustrative purposes only and should not be generalized to populations with different baseline mortality risks.

Using the pooled case-fatality rate of 22% as an illustrative pre-test probability, a positive IL-10 result increased the post-test probability of fatal outcome to 49.3%, whereas a negative result decreased it to 7.7% (**Figure 9**). The Fagan nomogram is presented for illustration only. Given the small evidence base, heterogeneous *post hoc* thresholds, potential methodological bias, and use of a pre-test probability derived from the broader meta-analysis, these post-test probabilities should not guide clinical decisions or support implementation of IL-10 as a prognostic test.

## Discussion

This meta-analysis indicates that higher serum IL-10 levels are associated with mortality in patients with SFTSV infection, while non-survivors consistently show higher concentrations than survivors across the main, sensitivity, and study-design subgroup analyses. However, the clinical interpretation of this association should remain cautious. The certainty of evidence was rated as very low because prognostic confounding was inadequately controlled in many studies, between-study heterogeneity was extreme, assay methods and sampling times varied substantially, and funnel-plot asymmetry suggested possible small-study effects. Although the association remained positive when analyses were restricted to studies not requiring conversion of medians and quantiles, this sensitivity analysis included only four studies and did not reduce heterogeneity, supporting directional consistency rather than a stable estimate of effect magnitude.

Similarly, positive findings were observed in both prospective and retrospective cohorts, whereas the case-control evidence was limited to a single study. The 95% prediction interval crossed zero, further indicating that the association may not be reproducible across different populations and methodological settings. Study-level IL-10 concentration was not used for subgroup classification because no validated thresholds were available and the categories were highly sensitive to extreme values, while exploratory meta-regression showed no significant relationship between IL-10 concentration and effect size. Threshold-based prognostic-accuracy analyses suggested potential discriminatory value, but only three studies were available, and all used *post hoc* cut-off values without pre-specification or external validation, resulting in a high risk of bias. Taken together, these findings support serum IL-10 as a potential marker of poor prognosis in SFTSV infection, but the large pooled standardized mean difference should be regarded as an average association across heterogeneous studies rather than evidence of established clinical validity or a universally applicable prognostic threshold.

IL-10 is an important anti-inflammatory and immunoregulatory cytokine secreted by T cells, macrophages, and other immune cells. Under infectious and inflammatory conditions, it limits tissue injury by suppressing excessive production of pro-inflammatory mediators such as TNF-α and IL-6 (11, 36, 37). However, markedly elevated IL-10 may also indicate severe immune dysregulation. It may represent a compensatory response to an intense cytokine storm and thus reflect disease severity (10), while excessive IL-10 may suppress antiviral T-cell responses, impair viral clearance, and contribute to secondary immune dysfunction (38, 39). Experimental evidence further suggests that the SFTSV nonstructural protein NSs can interfere with interferon signaling and activate TPL2-related pathways that promote IL-10 production, thereby facilitating viral immune evasion and pathogenesis (16, 40, 41). Nevertheless, these mechanisms were not directly examined in the clinical studies included in this meta-analysis and should not be interpreted as establishing a causal role for IL-10 in fatal SFTS. Importantly, elevated IL-10 may simply reflect severe inflammation and immune dysregulation rather than provide prognostic information independent of established clinical predictors such as age, viral load, or other routine risk factors.

Two studies warrant methodological consideration because of their markedly divergent effect estimates. Zhao et al. (32) reported an exceptionally large SMD of 7.10, which was mainly attributable to the very small standard deviation observed in the survivor group rather than to an error in data extraction. Although the reported values were verified against the original publication, the reason for this unusually limited dispersion could not be determined and may relate to assay characteristics, patient selection, small sample size, or other unreported methodological factors. In contrast, Yu et al. (28) reported nearly identical IL-10 concentrations in non-survivors and survivors, resulting in an SMD close to zero. Although IL-10 was reported in ng/mL and converted to pg/mL for consistency, this conversion did not affect the SMD because the same scaling factor was applied to both groups. These contrasting findings illustrate the substantial clinical and methodological variability among the included studies and further limit the generalizability of the pooled effect estimate.

The meta-regression did not demonstrate a statistically significant linear association between study-level IL-10 concentration and the magnitude of the mortality-related effect. The apparent positive trend observed in the original analysis may have been disproportionately influenced by Zhao et al. (2020) (32), which reported an unusually large SMD. Meta-regression based on only 12 studies has limited power to detect moderator effects and is particularly sensitive to influential observations. In addition, because the predictor represented an aggregated study-level value, the analysis is susceptible to ecological bias and cannot establish a patient-level concentration-response relationship.

This study has several limitations. First, substantial between-study heterogeneity was observed, and the 95% prediction interval crossed zero, indicating that both the direction and magnitude of the association may vary across future studies. This variability likely reflects differences in study design, patient characteristics, disease severity, sampling time, assay platforms, reporting units, and clinical management. Particularly, heterogeneity of sampling time was noticed because one study adopted peak IL-10 concentrations measured between days 5 and 15 after symptom onset, whereas the remaining studies used admission or early hospitalization values. Peak and baseline cytokine concentrations are not comparable, and their combined use may have introduced additional heterogeneity. Besides, four primary studies did not report the laboratory assay used to measure IL-10, limiting assessment of methodological comparability and potentially contributing to between-study heterogeneity. Simultaneously, 8 of the 12 included studies reported IL-10 concentrations as medians and interquartile ranges, requiring conversion to means and standard deviations. Because IL-10 concentrations may follow markedly right-skewed distributions, these transformations may not accurately reflect the original data and could introduce additional uncertainty or contribute to between-study heterogeneity. Although a sensitivity analysis restricted to the four studies directly reporting mean ± SD values was performed, the potential bias arising from data conversion could not be fully excluded. Although subgroup analyses were conducted, incomplete reporting in the original studies precluded more detailed evaluation according to assay method, comorbidities, treatment, disease stage, or other clinically relevant factors.

Second, funnel-plot asymmetry and Egger’s test suggested possible small-study effects; however, given the extreme heterogeneity, this pattern may also arise from methodological and clinical differences rather than selective publication alone. Publication bias therefore cannot be confirmed, and the exploratory trim-and-fill results should be interpreted cautiously because this method is unreliable when heterogeneity is substantial. Third, most included studies were observational, preventing causal inference as to whether elevated IL-10 contributes directly to mortality or primarily reflects greater disease severity. Residual confounding is also likely, as most studies did not adequately adjust for disease severity, viral load, organ dysfunction, timing of presentation, or other prognostic factors. Only three studies reported adjusted estimates, and differences in covariate selection and statistical modeling prevented a meaningful pooled adjusted analysis. In addition, the meta-regression included only 12 studies and was underpowered, sensitive to extreme observations, and vulnerable to ecological bias because moderators were assessed at the study rather than patient level.

Finally, the prognostic accuracy analysis was based on only three studies and should therefore be considered exploratory, particularly because the proposed IL-10 thresholds were not externally validated. The Fagan nomogram also used the pooled case-fatality rate only as an illustrative pre-test probability, while post-test probabilities are prevalence dependent and may differ substantially across populations and clinical settings. Large multicenter prospective studies using standardized IL-10 measurement, prespecified sampling protocols and thresholds, and comprehensive adjustment for established clinical confounders are therefore required before IL-10 can be considered a reliable prognostic biomarker in SFTS.

## Conclusion

This systematic review and meta-analysis indicated that elevated serum IL-10 levels were associated with mortality in patients with SFTS. However, confidence in the pooled estimate is substantially limited by residual confounding, extreme between-study heterogeneity, assay and sampling variability, possible small-study effects, limited geographic generalizability, and the small number of studies evaluating prognostic accuracy. IL-10 should therefore be regarded as a candidate prognostic biomarker reflecting inflammation and immune dysregulation rather than a validated stand-alone tool for routine clinical risk stratification. Its clinical utility requires confirmation in large prospective multicenter cohorts using standardized sampling protocols and assay platforms, predefined and externally validated cut-off values, and assessment of its incremental predictive value beyond established clinical risk factors.

## Data Availability

This systematic review protocol was registered in the International Prospective Register of Systematic Reviews (PROSPERO; registration No. CRD420251149587). The protocol is publicly accessible through PROSPERO via https://www.crd.york.ac.uk/prospero/.
