# Supplementary material for: Serum interleukin-10 levels and mortality in severe fever with thrombocytopenia syndrome: a systematic review and meta-analysis
Source: Front Immunol. 2026 Aug 7;17:1903694. doi: 10.3389/fimmu.2026.1903694 (PMC13493291; doi:10.3389/fimmu.2026.1903694)
Supplement: Supplementary file 1 [file DataSheet1.docx]

**Serum interleukin-10 levels and mortality in severe fever with thrombocytopenia syndrome: a systematic review and meta-analysis**

Yanan Yao^1^, Zanzan Sun^1^, Jianguo Zhang^1,*^, Zhimin Tao^1, 2, *^

^1^Department of Emergency Medicine, The Affiliated Hospital, Jiangsu University, Zhenjiang, Jiangsu 212001, China

^2^Jiangsu Key Laboratory of Medical Science and Laboratory Medicine, Department of Laboratory Medicine, School of Medicine, Jiangsu University, Zhenjiang, Jiangsu 212013, China

^*^Correspondence should be addressed to:

Jianguo Zhang: [1000011431@ujs.edu.cn](mailto:1000011431@ujs.edu.cn)

Zhimin Tao: [jsutao@ujs.edu.cn](mailto:jsutao@ujs.edu.cn)

**Search Strategy**

The systematic search was conducted in the following databases up to October 11, 2025:

1. PubMed (National Center for Biotechnology Information)

2. Embase (Elsevier)

3. Web of Science

4. CNKI

5. Wanfang Data

6. CQVIP

A combination of Medical Subject Headings (MeSH) and free-text terms was used.

The search strategy for PubMed is as follows:

1. “Severe Fever with Thrombocytopenia Syndrome” (All Fields) OR “Severe Fever with Thrombocytopenia Syndrome Bunyavirus Infection” (All Fields) OR “SFTS virus infection” (All Fields) OR “Infection, SFTS Virus” (All Fields) OR “SFTS Virus Infections” (All Fields) OR “Virus Infection, SFTS” (All Fields)
2. [“Interleukin 10” (All Fields) OR “CSIF-10” (All Fields) OR “IL-10” (All Fields) OR “IL 10” (All Fields)](https://www.webofscience.com/wos/woscc/summary/1534d229-34f3-43f2-afec-c9286ede3dc2-017a86a943/relevance/1)

3. #1 AND #2

4.Limits: No filters applied (All fields; no language, human/animal, or date restrictions)

The search strategy for Embase is as follows:

1. “severe fever with thrombocytopenia syndrome”/exp OR “severe fever with thrombocytopenia syndrome virus infection” OR “SFTS (severe fever with thrombocytopenia syndrome)”/exp OR “SFTS (severe fever with thrombocytopenia syndrome)” OR “SFTSV infection”/exp
2. “il 10” OR “il-10” OR “interleukin-10” OR “interleukin 10”

3. #1 AND #2

4. Filters: None (i.e., no language, date, species, or document type limits applied)

The search strategy for Web of Science is as follows:

1. TS=((“severe fever with thrombocytopenia syndrome virus infection”) OR (“SFTS (severe fever with thrombocytopenia syndrome)”) OR (“SFTS virus infection”) OR (“SFTSV infection”) OR (“severe fever with thrombocytopenia syndrome”))
2. TS=((“csii”) OR (“cytokine synthesis inhibitory factor”) OR (“il 10”) OR (“il-10”) OR (“interleukin-10”) OR (“interleukin 10”))
3. #1 AND #2

Please note that for the Chinese databases, the original Chinese search expressions are retained to reproduce the queries as entered. Each Chinese expression corresponds directly to the English translation provided immediately below it.

The search strategy for CNKI is as follows:

1. 主题=(发热伴血小板减少综合征+新型布尼亚病毒)

[Translation: Topic = (severe fever with thrombocytopenia syndrome+ novel bunyavirus)]

2. 主题=(白细胞介素-10+IL-10)

[Translation: Topic = (interleukin-10+IL-10)]

3. #1 AND #2

4. Filters: None (i.e., no language, date, species, only journal articles)

The search strategy for Wanfang Data is as follows:

1. 主题=(“发热伴血小板减少综合征” OR “新型布尼亚病毒”)

[Translation: Topic = (“severe fever with thrombocytopenia syndrome” OR “novel bunyavirus”)]

2. 主题=(“白细胞介素-10” OR “IL-10”)

[Translation: Topic = (“interleukin-10” OR “IL-10”)]

3. #1 AND #2
4. Filters: None (i.e., no language, date, species, only journal articles)

The search strategy for VIP Database is as follows:

1. 任意字段=(“发热伴血小板减少综合征” OR “新型布尼亚病毒”)

[Translation: Any item = (“severe fever with thrombocytopenia syndrome” OR “novel bunyavirus”)]

2. 任意字段=(“白细胞介素-10” OR “IL-10”)

[Translation: Any item = (“interleukin-10” OR “IL-10”)]

3. #1 AND #2

4. Filters: None (i.e., no language, date, species, only journal articles)

**Supplementary Table S1. Original IL-10 data formats and statistical conversion procedures in the included studies.** IL-10 concentrations reported as medians with IQRs or ranges were converted to estimated means and SDs using the method of Wan et al. (2014) and Luo et al. (2018). For studies reporting means and SEs, SDs were calculated using SD=SE×n^1/2^ (Higgins JP, et al. (2019)). No conversion was performed for studies directly reporting means and SDs.

| **Study** | **Non-survivors (original data)****, pg/mL** | **Survivors (original data), pg/mL** | **Original form** | **Conversion** | **Non-survivors (mean ± SD), pg/mL** | **Survivors (mean ± SD), pg/mL** |
| --- | --- | --- | --- | --- | --- | --- |
| Yoo et al. 2021 | 42.90 (8.20-145.20) | 8.80 (0.0-72.30) | Median+Range | Formula A | 59.18±50.20 | 13.78±16.29 |
| Zhang et al. 2012 | 86.30 (35.4-121.20) | 0.30 (0.30-24.90) | Median+IQR | Formula B | 80.31±76.66 | 9.03±18.90 |
| Liu et al. 2025 | 97.90 (50.50-236.50) | 20.20 (8.00-52.90) | Median+IQR | Formula B | 130.53±146.22 | 27.43±33.99 |
| Lu et al. 2024 | 96.70 (40.21-258.07) | 3.14 (1.79-8.27) | Median+IQR | Formula B | 134.54±175.26 | 4.47±4.89 |
| Ma et al. 2020 | 201.00 (80.8-342.5) | 19.90 (11.00-57.20) | Median+IQR | Formula B | 208.65±208.06 | 29.93±35.11 |
| Zhang et al. 2024 | 62.03 (14.67-120.10) | 8.90 (4.57-21.45) | Median+IQR | Formula B | 65.83±81.23 | 11.79±12.65 |
| Zang et al. 2024 | 145.00 (82.70-245.50) | 20.60 (10.05-49.50) | Median+IQR | Formula B | 158.54±124.76 | 27.05±29.56 |
| Yu et al. 2025 | 43300 (11700-82900) | 42600 (12600-75000) | Median+IQR | Formula B | 46140±54850 | 43440±46600 |

**Abbreviations**: IQR, interquartile range; SD, standard deviation; n, sample size; a, minimum value; b, maximum value; q1, first quartile (25^th^ percentile); q3, third quartile (75^th^ percentile); m, median (50^th^ percentile); Φ^-1^, inverse cumulative distribution function (quantile function) of the standard normal distribution.

**Formula A** (for median + range):

**Formula B** (for median + IQR):

**References:** 1) Wan X, et al. (2014) Estimating the sample mean and standard deviation from the sample size, median, range and/or interquartile range. BMC Med Res Methodol.14:135; 2) Luo D, et al. (2018) Optimally estimating the sample mean from the sample size, median, mid-range, and/or mid-quartile range. Stat Methods Med Res. 27:1785; 3) Higgins JP, et al. (2019) Choosing effect measures and computing estimates of effect. Cochrane Handbook for Systematic Reviews of Interventions,143-176.

**Supplementary Table S2. Characteristics of the studies included in the meta-analysis.** The table summarizes the country, IL-10 reporting unit, age and male proportion of non-survivors and survivors, timing of blood collection, specimen type, IL-10 detection method, and principal findings of the 12 included studies. Age is presented as median (interquartile range), mean (standard deviation), or as originally reported in each study. All studies measured IL-10 in serum. A dash indicates that the relevant information was not reported. CBA, cytometric bead array; IL-10, interleukin-10; NS, non-survivors; S, survivors; SD, standard deviation; SFTS, severe fever with thrombocytopenia syndrome.

| **Study** | **Country** | **IL-10 unit** | **Age/Male (%)** | **Sampling Timing** | **Specimen** | **Detection Method** | **Key Finding** |
| --- | --- | --- | --- | --- | --- | --- | --- |
| Yoo et al. 2021 | Korea | pg/mL | NS: 72.0 (62.3-81.9) / 85.7%; S: 61.4 (47.0-75.8) / 54.3% | At admission | Serum | Flow cytometry-CBA | IL-10 significantly higher in non-survivors than survivors |
| Li et al. 2014 | China | pg/mL | NS: 70.0 (58.0-75.0) / 52.4%; S: 58.0 (33.0-78.0) / 66.7% | Days 5-15 post-onset (peak) | Serum | Flow cytometry-CBA | IL-10 persistently elevated in deceased patients but declined in survivors |
| Zhang et al. 2012 | China | pg/mL | NS: 52.9 (SD 8.9) / —; S: 62.1 (SD 9.6) / — | At admission | Serum | Multiplex bead-based immunoassay (Luminex) | IL-10 significantly higher in fatal cases than survivors |
| Liu et al. 2025 | China | pg/mL | NS: 65 (54.2-75.8) / 44.0%; S: 62.1 (49.4-74.7) / 47.0% | At admission | Serum | Automated immunoassay | IL-10 greater in non-survivors than survivors |
| Hou et al. 2024 | China | pg/mL | NS: 68 (60.0-71.0) / 45.9%; S: 61 (56.0-69.3) / 37.5% | At admission | Serum | Chemiluminescent immunoassay | IL-10 elevated in non-survivors versus survivors |
| Yu et al. 2025 | China | ng/mL | NS: 71.6 (61.6-81.6) / 52.6%; S: 65.7 (55.5-75.9) / 50.3% | At admission | Serum | — | No significant difference in IL-10 between groups |
| Lu et al. 2024 | China | ng/L | NS: 67.5 (57.5-73.3) / 61.1%; S: 64.5 (54.0-72.0) / 47.6% | First week after admission | Serum | — | IL-10 significantly higher in deceased than surviving group |
| Zhao et al. 2020 | China | pg/mL | — / —; S: — / — | At admission | Serum | Liquid-phase chip technology (Luminex) | IL-10 significantly higher in deceased patients |
| Ma et al. 2020 | China | pg/mL | NS: 63.1 (54.6–71.6) / —; S: 60.7 (50.9–70.5) / — | Early stage of hospitalization | Serum | — | IL-10 higher in death group than survival group |
| Liu et al. 2020 | China | pg/mL | NS: 64.3 / 47.5%; S: 64.2 / 52.9% | At admission | Serum | Flow cytometry | IL-10 significantly higher in death group than recovery group |
| Zhang et al. 2024 | China | pg/mL | NS: 65.8 (59.1–72.6) / 44.7%; S: 60.0 (50.2–69.8) / 37.3% | At admission | Serum | Flow cytometry (immunofluorescence) | IL-10 significantly higher in death than survival group |
| Zang et al. 2024 | China | ng/L | NS: 69.0 (64.0–75.0) / —; S: 60.0 (52.0–68.0) / — | At admission | Serum | — | IL-10 significantly higher in deceased than surviving group. |

**Supplementary Table S3. GRADE-based certainty-of-evidence assessment for the association between serum IL-10 levels and mortality in SFTS.** The table presents the initial certainty, judgments for risk of bias, inconsistency, indirectness, imprecision, and possible small-study effects, together with a detailed GRADE summary that lists the basis for each downgrade.

| **Outcome** | **Study design** | **Initial certainty** | **Risk of bias^1^** | **Inconsistency^2^** | **Indirectness^3^** | **Imprecision^4^** | **Possible small-study effects^5^** | **Final certainty** |
| --- | --- | --- | --- | --- | --- | --- | --- | --- |
| IL-10 level associated with poor prognosis | Observational cohort studies | Low | Serious (−1) | Very serious (−2) | Very serious (−2) | Very serious (−2) | Likely  (−1) | Very low |

**Reasons for downgrade**

^1^QUIPS assessment showed high risk of inadequate confounding adjustment across the included observational cohort studies.

^2^Substantial heterogeneity was observed (*I*²=94.6%); the 95% prediction interval crossed the null effect value, indicating inconsistent effect estimates among individual studies.

^3^There was major inconsistency in detection platforms, blood sampling timing and diagnostic cut-off thresholds for IL-10 detection. ^4^All enrolled patients were recruited from hospitals in Eastern China and South Korea, limiting generalizability to other populations and medical settings. The prediction confidence interval crosses zero, and the prognostic accuracy analysis is based on only three studies, leading to imprecise effect estimates.

^5^Egger’s regression test demonstrated statistically significant publication bias; visual inspection of funnel plot revealed obvious asymmetry.

**Footnote:** Certainty of evidence cannot be downgraded below ‘very low’. Where the cumulative domain-specific downgrades exceed the numerical range of the GRADE scale, the additional downgrades indicate multiple serious methodological concerns and do not represent further reductions below the ‘very low’ category.

**Supplementary Figure S1**. **Detailed QUIPS risk of bias assessment for each included study.** QUIPS comprises six domains: D1 (study participation), D2 (study attrition), D3 (prognostic factor measurement), D4 (outcome measurement), D5 (study confounding), and D6 (statistical analysis and reporting). Each domain was rated as low, moderate, or high risk of bias. For D1, D4, and D6, all 12 studies were rated as low risk. For D2, 11 studies were low risk and 1 was moderate risk. For D3, 7 studies were low risk and 5 were moderate risk. For D5, all 12 studies were rated as moderate risk, because most studies did not adequately adjust for important confounders (e.g., age, comorbidities, disease severity at admission, and coinfections). Consequently, the overall risk of bias was rated as moderate for all 12 studies, driven by the consistent moderate rating in D5 and the additional moderate ratings in D2 (one study) and D3 (five studies). QUIPS, Quality In Prognosis Studies.

| **Study** | **Study participation** | **Study attrition** | **Prognostic factor measurement** | **Outcome measurement** | **Study confounding** | **Statistical analysis and reporting** | **Overall** |
| --- | --- | --- | --- | --- | --- | --- | --- |
| Yoo et al. 2021 | Low | Moderate | Low | Low | Moderate | Low | Moderate |
| Zhang et al. 2012 | Low | Low | Low | Low | Moderate | Low | Moderate |
| Liu et al. 2025 | Low | Low | Low | Low | Moderate | Low | Moderate |
| Lu et al. 2024 | Low | Low | Moderate | Low | Moderate | Low | Moderate |
| Ma et al. 2020 | Low | Low | Moderate | Low | Moderate | Low | Moderate |
| Zhang et al. 2024 | Low | Low | Low | Low | Moderate | Low | Moderate |
| Li et al. 2014 | Low | Low | Moderate | Low | Moderate | Low | Moderate |
| Hou et al. 2024 | Low | Low | Low | Low | Moderate | Low | Moderate |
| Zhao et al. 2020 | Low | Low | Low | Low | Moderate | Low | Moderate |
| Liu et al. 2020 | Low | Low | Low | Low | Moderate | Low | Moderate |
| Zang et al. 2024 | Low | Low | Moderate | Low | Moderate | Low | Moderate |
| Yu et al. 2025 | Low | Low | Moderate | Low | Moderate | Low | Moderate |

**Supplementary Figure S2. QUADAS-2 risk of bias summary.** QUADAS-2 risk of bias assessment for the three studies contributing to the prognostic accuracy analysis. QUADAS‑2 comprises four domains: D1 (patient selection), D2 (index test), D3 (reference standard), and D4 (flow and timing). Each domain was rated as low, high, or unclear risk of bias, with the overall rating determined by the highest-risk domain. All three studies were rated as low risk in D1, D3, and D4, but high risk in D2 (index test) because their IL‑10 cut‑off values were derived post hoc using ROC analysis rather than being prespecified. Consequently, the overall risk of bias was rated as high for all three studies. QUADAS‑2, Quality Assessment of Diagnostic Accuracy Studies‑2; ROC, receiver operating characteristic.

| **Study** | **Patient Selection** | **Index Test** | **Reference Standard** | **Flow and Timing** | **Overall** |
| --- | --- | --- | --- | --- | --- |
| Liu et al. 2025 | Low | High | Low | Low | High |
| Hou et al. 2024 | Low | High | Low | Low | High |
| Zhang et al. 2024 | Low | High | Low | Low | High |

**Supplementary Figure S3. Results of trim-and-fill analysis.** The funnel plot displays study-specific standardized mean differences (SMDs) against their standard errors. Open circles represent the observed studies. The horizontal line indicates the pooled SMD, and the diagonal lines indicate the pseudo 95% confidence limits. The trim-and-fill procedure did not identify any studies for imputation (*k*=0); therefore, the pooled estimate within this analysis remained unchanged at an SMD of 2.11 (95% CI: 1.42-2.79). Because only 12 studies were included and between-study heterogeneity was extreme (*I*²=94%), the analysis should be interpreted cautiously and does not exclude publication bias or small-study effects. CI, confidence interval; SMD, standardized mean difference.

| **Model** | **n** | **Pooled SMD** | **95% CI** | **Heterogeneity Q** | **Iteration Result** |
| --- | --- | --- | --- | --- | --- |
| Original random-effects | 12 | 2.11 | [1.42, 2.79] | Q=202.38, df=11, P<0.01 | - |
| Trim-and-Fill Adjusted | 12 | 2.11 | [1.42, 2.79] | Q=202.38, df=11, P<0.01 | 0 |

**Supplementary Figure S4. Sensitivity analysis of directly reported studies (n=4).** The standardized mean difference (SMD, Hedges’ *g*) and 95% confidence interval (CI) for each study are represented by squares and horizontal lines, with the size of each square reflecting the study weight in the random‑effects model (DerSimonian-Laird method). This sensitivity analysis included only studies that reported IL-10 levels as means and SDs directly (Li et al., 2014; Liu et al., 2020; Hou et al., 2024; and Zhao et al., 2020), excluding the eight studies that required median‑to‑mean conversion using the Wan method. The pooled SMD was 3.61 (95%CI: 1.29-5.93), with extremely high heterogeneity (*I*²=97%). Although the direction of the association was consistent with the main analysis, the estimate was highly unstable and based on very few studies, two of which had significant methodological limitations (small sample size and case-control design). CI, confidence interval; DL, DerSimonian‑Laird; SMD, standardized mean difference.

**Supplementary Figure S5. Exploratory subgroup analysis by IL‑10 concentration category (post hoc).** Studies were classified into low, medium, and high concentration groups based on their mean IL‑10 levels relative to the overall mean (139.02 pg/mL) and SD (137.16 pg/mL) across ten studies: low (< mean−0.5*SD), medium (mean±0.5*SD), and high (> mean + 0.5*SD). This classification was derived post hoc and lacks biological or external validation; results should be interpreted with caution.

**Supplementary Figure S6. Meta-regression analysis of IL-10 concentration and mortality-related effect size.** Each circle represents an individual study (two influential studies including Yu et al. 2025 and Liu et al. 2020 were excluded), with the size of the circle reflecting the study weight. The predictor was the natural logarithm of the study-level mean IL-10 concentration in pg/mL. The fitted line shows its relationship with the standardized mean difference (SMD). No statistically significant association was identified (coefficient=0.73, 95% CI: −1.48-2.93; P=0.47). Because only 10 studies were included, the analysis was substantially underpowered and should be considered exploratory. CI, confidence interval; SMD, standardized mean difference.

| Index | Coefficient | Std. err. | t | P | 95%CI |
| --- | --- | --- | --- | --- | --- |
| Ln (IL-10 concentration) | 0.73 | 0.96 | 0.76 | 0.47 | [-1.48, 2.93] |
| Intercept | -1.28 | 4.60 | -0.28 | 0.79 | [-11.90, 9.33] |
